# Evaluation of a Fluorescence Immunoassay-Based IGRA for Latent Tuberculosis Diagnosis: A Simplified, Cost-Effective Alternative

**DOI:** 10.3390/microorganisms14030603

**Published:** 2026-03-09

**Authors:** Mohammad Khaja Mafij Uddin, Aar Rafi Mahmud, Afsana Akter Rupa, Ashabul Islam, Jahin Fairuj Oishi, Jannatul Ferdous, Rumana Nasrin, Syed Mohammad Mazidur Rahman, Senjuti Kabir, Shahriar Ahmed, Sayera Banu

**Affiliations:** 1 Infectious Diseases Division, International Centre for Diarrhoeal Disease Research, Dhaka 1212, Bangladesh; aar.mahmud@icddrb.org (A.R.M.); afsana.rupa@icddrb.org (A.A.R.); jahinoishi7@gmail.com (J.F.O.); jannatul.ferdous3@icddrb.org (J.F.); rumana.nasrin@icddrb.org (R.N.); smmazidur@icddrb.org (S.M.M.R.); sbanu@icddrb.org (S.B.); 2 Department of Mathematics and Natural Sciences, BRAC University, Dhaka 1212, Bangladesh; ashabulzudan@gmail.com

**Keywords:** latent tuberculosis, ELISA, lateral flow assay, ichroma IGRA-TB, QFT-plus

## Abstract

Approximately 25% of the global population is estimated to have latent tuberculosis infection (LTBI), with a 5–10% lifetime risk of progression to active disease. Although interferon-gamma release assays (IGRAs) are widely used for LTBI diagnosis, their high cost and operational complexity limit large-scale implementation in resource-limited settings. This study evaluated the diagnostic performance of a low-complexity, rapid, fluorescence-based point-of-care assay, ichroma IGRA-TB, for LTBI detection. A total of 300 participants enrolled at TB Screening and Treatment Centers and the Dhaka Hospital of icddr,b were categorized as healthy controls (*n* = 130), household contacts of TB patients (*n* = 70), GeneXpert MTB/RIF Ultra-positive active TB patients (*n* = 80), or individuals with a previous history of TB (*n* = 20). ichroma IGRA-TB was compared with QuantiFERON-TB Gold Plus (QFT-Plus) across all groups. Overall agreement between ichroma IGRA-TB and QFT-Plus was 91.9%, with a Cohen’s kappa of 0.83, indicating almost perfect concordance. Using culture as a surrogate reference standard, QFT-Plus demonstrated higher sensitivity (74.6%) than ichroma IGRA-TB (69.0%). Overall, ichroma IGRA-TB demonstrates high agreement with QFT-Plus and acceptable sensitivity, supporting its potential as a near-point-of-care tool for LTBI screening in resource-constrained settings.

## 1. Introduction

Tuberculosis (TB), caused by *Mycobacterium tuberculosis* (MTB), remains one of the leading causes of death from infectious disease agents globally, with an estimated 10.7 million people falling ill and more than a million people losing their lives in 2024 [1]. Latent tuberculosis infection (LTBI) is defined by persistent immune response to MTB antigens without any clinical signs or radiological evidence of active disease [2]. About 25% of the total population has LTBI worldwide [3]. Around 5–10% of LTBI cases develop active TB disease, while the remaining 90–95% remain in the latent stage without any symptom [4]. People with LTBI are at risk of developing active TB disease in their lifetime, mostly within the first five years [4,5]. Therefore, early detection and treatment of LTBI are crucial for effective TB control and for reducing TB incidence. However, the actual global burden of LTBI remains uncertain due to a lack of universally recognized gold-standard diagnostic tests [2]. The two most widely used tests are the tuberculin skin test (TST) and interferon-gamma (IFN-γ) release assay (IGRA). The TST is a traditional, low-cost test that measures the delayed-type skin reaction to purified protein derivative (PPD) following intradermal injection [6,7,8]. However, it can show false positive results due to previous BCG vaccination, or exposure to nontuberculous mycobacteria (NTM), while immunocompromised conditions are associated with false negative results owing to impaired cell-mediated immunity [8]. Moreover, the skin-based tests have lower sensitivity [9]. QuantiFERON-TB Gold Plus (QFT-Plus; Qiagen, Hilden, Germany) is a widely used IGRA. It uses an enzyme-linked immunosorbent assay (ELISA) to measure IFN-γ released by both CD4+ and CD8+ T-cells [2,10]. Although this assay provides higher specificity, it is expensive, time-consuming, labor-intensive, and needs sophisticated laboratory setups [11].

In countries with a high TB burden and resource-constrained settings like Bangladesh and Southeast Asia [1], these barriers limit the wider implementation of IGRAs for LTBI detection. The ichroma IGRA-TB, a novel lateral flow immunoassay (LFA), has been developed by Boditech Med Inc. (Chuncheon-si, Republic of Korea) and might overcome several limitations of current IGRAs [10]. The ichroma IGRA-TB employs a sandwich immunodetection principle, in which antigen–antibody complexes formed in the sample are captured on a nitrocellulose membrane. The resulting fluorescence signal, proportional to the antigen concentration, is quantified by the ichroma analyzer to determine positive or negative latent TB infection results. It consists of three components: a Nil tube used as the negative control, a TB antigen tube containing MTB-specific antigens (ESAT-6 and CFP-10), and a mitogen tube used as the positive control. It uses a lateral flow cartridge to detect IFN-γ, with quantitative measurement performed by a portable fluorescence analyzer (ichroma™ II reader) providing results within 20 min. Compared to the QFT-Plus IFN-γ ELISA, the ichroma IGRA-TB offers a relatively faster turnaround time, minimal technical requirements, and greater accessibility for decentralized testing. Moreover, its time-resolved fluorescence-based detection system enhances analytical sensitivity compared to conventional gold particle or fluorophore-based rapid assays [10]. Limited data are available on easy to use and rapid LFA based IGRA-TB assays (Table 1). In this study, we aimed to evaluate the diagnostic performance of ichroma IGRA-TB in Bangladesh, a high-burden and resource-limited setting, by comparing it to the QFT-Plus assay across various population risk categories. The findings of this study provide evidence on the feasibility of a rapid, low-complexity IGRA platform that may support decentralized LTBI screening and programmatic decision-making in high-burden, resource-limited settings.

## 2. Materials and Methods

### 2.1. Study Design and Participant Enrolment

We enrolled 300 individuals of different risk groups from the TB Screening and Treatment Centre (TBSTC) and Dhaka Hospital of icddr,b between 15 December 2024 and 25 August 2025. icddr,b has been conducting TB screening among presumptive TB patients through TBSTCs in Bangladesh since 2014. Presumptive pulmonary TB patients presenting at TBSTCs are verbally screened for signs and symptoms of TB. Based on the screening, symptomatic individuals are referred for TB diagnosis with state-of-the art chest X-ray and GeneXpert assay. Dhaka Hospital meets the urgent need to treat patients, particularly children, with severe diarrhoeal disease. Children from different categories were recruited from this hospital.

### 2.2. Determination of Categories and Number of Study Participants

Participants were divided into four different groups: group 1 (Healthy control; *n* = 130) comprised asymptomatic healthy individuals without a history of TB or known exposure to TB patients; group 2 (TB contacts; *n* = 70) included household or close contacts of TB patients or healthcare workers related to TB patient care; group 3 (Active TB; *n* = 80) consisted of newly diagnosed TB patients who had not yet started anti-TB treatment; and group 4 (TB history; *n* = 20) involved individuals with previously tested with TB and had completed a full course of treatment prior to enrolment.

### 2.3. Sample Size Determination Among Different Risk Groups

Sample size was calculated considering 5% Type I Error and 10% Marginal Error. For Group 1, 2, and 4, true disease status was unknown at the time of enrolment, and prevalence was assumed from our recently published study [12].

For Group 1, assuming 90% sensitivity for QFT-plus and 70% sensitivity for ichroma in detecting LTBI, a 29% prevalence of LTBI, and 80% power, the required sample size was 147 after Yates’ Continuity Correction. For Group 2, adjusting the prevalence to 37%, the required sample size was 115 after continuity correction. For Group 3, adjusting the prevalence to 60%, the required sample size was 71. In Group 3, monthly, around 800–900 TB presumptive patients visit the TBSTC facility of icddr,b, and among them, 10–13% of patients test positive for TB. We only enrolled patients who had been confirmed for TB into Group 3 of this study. We found IGRA assays to have 74.1–76.5% sensitivity vs. culture testing [12]. Assuming 75% sensitivity of the ichroma IGRA TB assay among confirmed active TB patients, 5% Type 1 Error, 10% Marginal error, and 100% prevalence (due to enrolment of only confirmed cases) and adjusting for continuity, we enrolled 80 confirmed active TB cases. Due to resource constraints, we recruited a total of 300 participants. Finally, we enrolled 130 healthy individuals in Group 1, 70 healthy contacts in Group 2, 80 confirmed active TB cases in Group 3, and 20 individuals with prior TB in Group 4.

### 2.4. Demographic and Clinical Data Collection

Demographic and clinical data were collected using a structured case record form at enrolment. Smoking status was defined as current use of any tobacco product at the time of recruitment. Diabetes mellitus was defined based on self-reported physician diagnosis or documented use of anti-diabetic medication. All data were collected prior to laboratory testing to avoid outcome-related bias.

### 2.5. Blood and Respiratory Sample Collection

A 7 mL venous blood sample was drawn from each participant, 3 mL of which was evenly transferred to ichroma IGRA-TB tubes (Nil, TB Ag, and mitogen) and the remaining 4 mL was distributed into a set of QFT-Plus tubes (Nil, TB1, TB2, and mitogen). The blood samples were kept in a sterile container at room temperature until transport to the laboratory. Under aseptic conditions, the tubes were then transferred to the laboratory within two hours of collection. For Group 3 (active TB) participants who tested positive for MTB via the Xpert MTB/RIF Ultra assay at the TBSTC, additional sputum samples were collected for culture. Participants were excluded if they were unable to provide samples, refused to provide consent, or had already initiated anti-TB treatment.

### 2.6. QuantiFERON-TB Gold Plus (QFT-Plus) and ichroma IGRA-TB Assay

After blood collection, the tubes were mixed properly by shaking the tubes ten times firmly; all tubes were incubated upright position at 37 °C for 20 h to ensure uniform antigen stimulation. After incubation, the tubes were centrifuged for 15 min at 3000 RCF. Approximately 300 μL of plasma was harvested and stored at 4 °C for three days and −80 °C for long-term storage. IFN-γ was measured from the plasma samples via ELISA (QFT-Plus) and LFA (ichroma IGRA-TB). Both assays were performed on the same day.

All procedures for both the ichroma IGRA-TB and QFT-Plus assays were performed following the manufacturers’ instructions [13,14]. For the ichroma IGRA-TB assay, plasma samples from Nil, TB Ag, and mitogen tubes were processed with the ichroma II analyzer in multi-test mode. In short, 100 μL of the detector diluent was added to the detector tube to make the detection buffer, after which 50 μL of plasma samples were added. Then 100 μL of the sample–buffer mixture was then loaded on the corresponding test cartridges and incubated at room temperature for 15 min. After that, cartridges were inserted serially into the ichroma II analyzer and the IFN-γ level (IU/mL) was measured automatically. Results were positive if the concentration of IFN-γ in the TB antigen tube minus the nil control was greater than or equal to 0.35 IU/mL and greater than or equal to 25% of the nil value. Results were considered negative if the IFN-γ level was less than 0.35 IU/mL or less than 25% of the nil. An indeterminate result was defined as a nil value greater than 8.0 IU/mL or when the antigen minus nil value was greater than or equal to 0.35 IU/mL but less than 25% of the nil.

### 2.7. Sample Processing for Culture

Sputum samples collected for Group 3 participants were transported to the Mycobacteriology Laboratory within 24 h for additional laboratory analysis. The samples were decontaminated using the standard N-Acetyl-L-Cysteine-Sodium Hydroxide (NALC-NaOH) method [15]. Following decontamination, the resulting sediment was resuspended in 1.0 mL of phosphate-buffered saline (PBS). Two loopfuls of sediments were inoculated onto Lowenstein–Jensen (L-J) slants and incubated at 37 °C for eight weeks to assess visible growth.

### 2.8. Statistical Analysis

Data analysis was conducted using R (version 4.5.1) with RStudio (Version 4.4.3, RStudio Team, 2023). Agreement between assays was evaluated by determining the positive percent agreement (PPA) and negative percent agreement (NPA) and Cohen’s kappa coefficient, based on 2 × 2 contingency table analysis. Chi-square or Fisher’s exact tests were used to assess any significant effect of diabetes or smoking on IGRA results. The chi-square test was also applied to evaluate whether significant differences occurred between ichroma IGRA-TB and QFT-Plus within study groups. Indeterminate results were excluded from the statistical analysis.

Spearman’s rank correlation was used to assess the association between QFT (TB1-Nil and TB2-Nil) and ichroma IFN-γ concentrations. Values were log-transformed after applying a constant shift to address skewness and zero/negative values. Linear regression was performed to evaluate proportional relationships between assays. Agreement was assessed using Bland–Altman analysis on log-transformed values, with mean bias and 95% limits of agreement calculated. Statistical significance was defined as *p* < 0.05 at a 95% confidence interval (CI).

## 3. Results

### 3.1. Clinical Features of Recruited Individuals

Baseline demographic and clinical characteristics of the enrolled participants are summarized in Table 2. Of the 300 participants, 162 (54.0%) were female, reflecting a modest female predominance across most study groups except Group 3. The majority of participants were aged 18–35 years (197/300, 65.7%). Overall, 19 participants (6.3%) had diabetes mellitus and 58 (19.3%) were current smokers. These variables are presented descriptively to characterize the study population and provide context for subsequent analyses assessing the influence of diabetes and smoking status on IGRA test performance.

### 3.2. Group-Based Analysis of QFT-Plus and ichroma IGRA-TB Outcomes

Table 3 presents the distribution of ichroma IGRA-TB and QFT-Plus results across the four study groups. In Group 1 (Healthy controls), ichroma IGRA-TB and QFT-Plus positivity rates were 23.1% (30/130) and 22.3% (29/130), respectively. Group 2 (TB contacts) showed positivity rates of 32.9% (23/70) for ichroma IGRA-TB and 31.4% (22/70) for QFT-Plus. Among Group 3 (Active TB patients), 65% (52/80) were positive according to ichroma IGRA-TB and 70% (56/80) according to QFT-Plus. Notably, all indeterminate results (observed only in QFT-Plus, Group 3) tested negative with ichroma IGRA-TB; these indeterminate results were excluded from the analysis. In Group 4 (History of TB), ichroma IGRA-TB and QFT-Plus positivity rates were 70% (14/20) and 60% (12/20), respectively. Overall, both assays demonstrated similar positivity across the study population (*n* = 119, 39.7%).

### 3.3. Comparison of ichroma IGRA-TB and QFT-Plus Results

Overall, the agreement between ichroma IGRA-TB and QFT-Plus was 91.9% (88.2–94.5%), with an overall PPA of 89.9% (83.2–94.1%) and an NPA of 93.2% (88.5–96.1%) (Table 4). Among healthy individuals (Group 1), the ichroma IGRA-TB test showed a PPA of 86.2% (69.4–94.5%) and an NPA of 95.0% (88.9–97.9%), with an overall agreement of 93.1% (87.4–96.3%) and a Cohen’s kappa of 0.80. The highest concordance was observed among TB contacts (Group 2), with an overall agreement of 95.7% (88.1–98.5%). It showed a PPA of 95.5% (78.2–99.2%) and an NPA of 95.8% (86.0–98.8%), yielding a Cohen’s kappa of 0.90, indicative of almost perfect agreement. In the newly diagnosed TB group (Group 3), the PPA, NPA, overall agreement, and kappa value were 87.5% (76.4–93.8%), 84.2% (62.4–94.5%), 86.7% (77.2–92.6%), and 0.67, respectively, and lower than those of any other groups. For previously treated TB cases (Group 4), the assay achieved 100.0% PPA (75.8–100.0%) and 75.0% NPA (40.9–92.9%), with an overall agreement of 90.0% (69.9–97.2%) and kappa value of 0.78.

### 3.4. Performance of QFT-Plus and ichroma IGRA-TB Assay Compared to Culture as Gold Standard

Excluding five indeterminate results, Group 3 comprised 75 samples, among which 71 were culture-positive and 4 were culture-negative. Among the 71 culture-positive individuals, 53 (74.6%) were positive on QFT-Plus and 49 (69.0%) on the ichroma IGRA-TB assay (Table 5). Compared to culture as the gold standard, QFT-Plus showed a sensitivity of (74.6%) whereas ichroma IGRA-TB showed 69.0%; PPV values were 94.6% for QFT-Plus vs. 94.2% for ichroma IGRA-TB. Due to the limited number culture-negative samples, specificity and negative predictive values did not reflect the actual scenario and hence were omitted from the analysis. The overall agreement with culture results was 72% for QFT-Plus and 66.7% for ichroma IGRA-TB.

### 3.5. Association of Risk Factors with Performance of QFT-Plus and ichroma IGRA-TB Assay Among Culture-Confirmed Patients

Of the 71 culture-positive individuals, QFT-Plus showed a positive result for 53 cases, of which 9 were diabetic and 44 were non-diabetic (Table 6). QFT-Plus positivity rates showed no significant difference between diabetic and non-diabetic participants (75% vs. 74.5%, *p* = 1.00), indicating that diabetes had no measurable effect on assay performance. However, patients with a smoking history had significantly lower positivity rates than non-smokers (56% vs. 84.7%, *p* = 0.017). On the other hand, ichroma IGRA-TB was positive for 49 out 71 culture-positive cases. For ichroma IGRA-TB, the positivity rate was lower in diabetic individuals than in non-diabetics (50% vs. 72.8%), although this difference was insignificant (*p* = 0.22). In contrast, similar to QFT-Plus, smoking status significantly influenced ichroma IGRA-TB results: smokers had a lower positivity rate compared with non-smokers (48% vs. 80.4%, *p* = 0.01).

### 3.6. Correlation and Agreement Between QFT and ichroma IFN-γ Measurements

**Figure 1:**
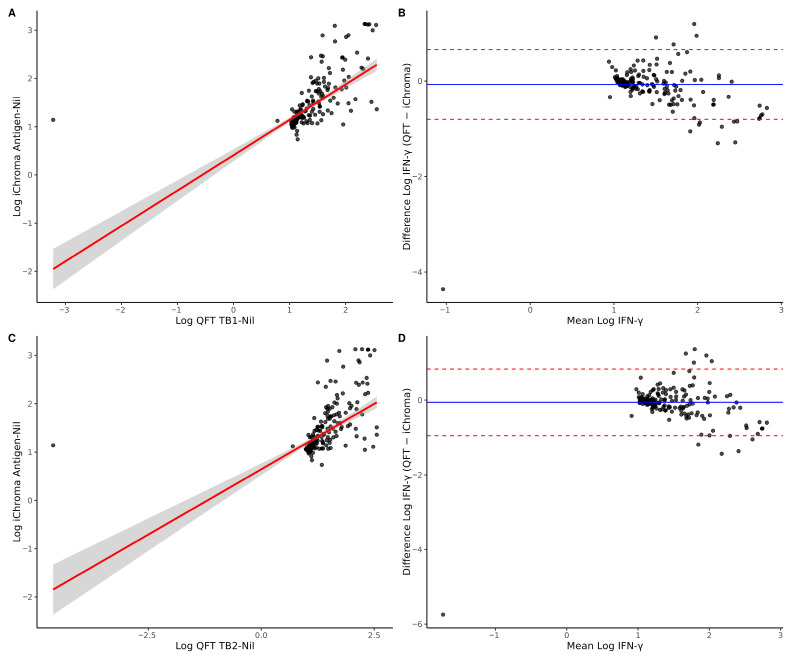
Quantitative comparison between QFT and ichroma IFN-γ measurements: (**A**) a scatter plot showing the relationship between ichroma antigen-Nil and QFT TB1-Nil values on a log-transformed scale, with the linear regression line and 95% confidence interval; (**B**) the Bland–Altman plot for ichroma antigen-Nil and QFT TB1-Nil values (log-transformed), showing mean bias (solid line) and 95% limits of agreement (dashed lines); (**C**) a scatter plot showing the relationship between ichroma antigen-Nil and QFT TB2-Nil values on a log-transformed scale, with the linear regression line and 95% confidence interval; (**D**) the Bland–Altman plot for ichroma antigen-Nil and QFT TB2-Nil values (log-transformed), showing mean bias (solid line) and 95% limits of agreement (dashed lines).

Spearman’s rank correlation demonstrated a strong positive association between QFT TB1-Nil and ichroma IFN-γ concentrations (ρ = 0.794, *p* < 0.001), as well as between QFT TB2-Nil and ichroma IFN-γ (ρ = 0.792, *p* < 0.001) (Figure 1A,C). Linear regression analysis of log-transformed values revealed a significant relationship between TB1 and ichroma (R^2^ = 0.45, *p* < 0.001), with a slope of 0.73, indicating a moderate proportional relationship. For TB2, the association was weaker (R^2^ = 0.33, *p* < 0.001), with a slope of 0.54. Bland–Altman analysis demonstrated minimal mean bias for TB1 (−0.07) and TB2 (−0.06) (Figure 1B,D), suggesting no substantial systematic difference between assays. However, the relatively wide 95% limits of agreement indicate variability between methods, with TB2 exhibiting slightly broader limits than TB1.

## 4. Discussion

In this stud, we assessed the diagnostic performance of the ichroma IGRA-TB assay in detecting LTBI compared with the widely used QFT-Plus assay in a high-TB-burden setting. The findings of this study demonstrate that ichroma IGRA-TB shows high overall concordance with QFT-Plus across different groups of participants.

In this study. the overall agreement of ichroma IGRA-TB with QFT-Plus was 91.9% (Cohen’s kappa 0.83). Multiple studies conducted in South Korea reported similar findings, with agreement ranging from 89.2% to 95.2%. [10,11]. These consistent results across different populations, including healthcare workers, healthy individuals, and immunocompromised patients, indicate the diagnostics value of ichroma IGRA-TB along with widely established QFT-Plus assay.

Because there is no definitive gold-standard test, it is challenging to assess the sensitivity and specificity of LTBI diagnostics. The specificity of such tests for LTBI is mainly determined from low-TB-burden countries. In this study, we used the QFT-Plus assay as the comparator to determine the specificity of the investigated assay (ichroma IGRA-TB). This approach might not be useful for high-burden settings like Bangladesh. Among the healthy individuals (Group 1) with no symptoms or no history of TB contacts, QFT-Plus and ichroma IGRA-TB showed similar positivity rates of 22.3% and 23.1%, respectively. Our study findings reflect the global LTBI prevalence of 24.8% reported in a recent meta-analysis based on IGRAs [16]. Similar findings were reported in our previous study where QFT-Plus and another ELISA-based TB IGRA (TB Feron) showed positivity rates of 26.1% and 29.1%, respectively, among the healthy control group [12]. Our current findings represent the closest estimation of TB infection prevalence (30.8%) in Southeast Asia [3].

Among TB contacts (Group 2), QFT-Plus and ichroma IGRA-TB showed positivity rates of 31.4% and 32.9% respectively. These results align with our recently published study, which reported a 38% QFT-Plus positivity rate [12].

In Group 4 (individuals with previous history of TB), QFT-Plus and ichroma IGRA-TB positivity rates were 60.0% and, 70.0%, respectively. In our previous study, we also found similar positivity rates with the QFT-Plus assay [12]. Other studies reported that levels of interferon-gamma in IGRA tests may increase, decrease, or remain unchanged after treatment completion [17,18].

In this study, QFT-Plus yielded indeterminate results for five samples (1.7%), while ichroma IGRA-TB yielded all negative results. According to the assay performances, mitogen tubes served as a positive control in both assays and represented the patient’s immune system by producing interferon-gamma. Subtraction of nil values from mitogen should be >5.0 IU/mL for a valid test result (either positive or negative). Careful observation suggests that the average mitogen value of these indeterminate samples was lower in the QFT-Plus assay compared to ichroma IGRA-TB (0.35 vs. 6.4). This might be due to the specific mitogen tube of the QFT-Plus assay for these samples. Moreover, the assay procedure may also have impacted the test results.

In this study, 12 samples were positive in the QFT-Plus assay but negative in ichroma IGRA-TB, while an additional 12 samples were found to be positive in ichroma IGRA-TB but negative in QFT-Plus. Discordance among ichroma IGRA-TB positives but QFT-Plus negatives primarily occurred due to the quantitative values close to the cutoff (0.39 to 0.82 IU/mL). A previous study also reported discordant results with different IGRAs where the quantitative value of cutoff was 0.2 to 0.7 IU/mL [10].

QFT-Plus showed slightly higher sensitivity (74.6%) than ichroma IGRA-TB (69.0%) when compared with the culture results of the active TB group. In the active TB group (Group 3), QFT-Plus showed a slightly higher positivity rate than ichroma IGRA-TB, whereas in the group with a previous history of TB, ichroma IGRA-TB demonstrated a higher positivity rate compared to QFT-Plus. These differences were modest and did not substantially affect the overall concordance between the two assays. These values were lower than the pooled sensitivity reported in meta-analyses for QFT-Plus (92.6%) [19] and ichroma IGRA-TB (80.56%) [20], which may reflect differences in cohort characteristics, including immune status. This result is consistent with IGRA performance among immunocompromised individuals, where sensitivity may fall to approximately 70% [21]. This indicates the influence of host immune response on IFN-γ-based diagnostics.

The comparatively lower sensitivity observed for ichroma IGRA-TB in active TB patients should be interpreted with caution, as no additional analyses were performed to determine the underlying cause. Possible explanations include differences in assay platforms and detection thresholds between ELISA-based QFT-Plus and LFA-based ichroma IGRA-TB, as well as immune dysregulation in active TB leading to borderline IFN-γ responses. Importantly, the difference was not statistically significant, suggesting that the observed variation likely reflects biological variability and sample size rather than a true performance disparity between the assays.

Among culture-positive individuals, we evaluated whether risk factors such as diabetes or smoking had any effect on the positivity of both QFT-Plus and ichroma IGRA-TB. The positivity rate for QFT-Plus was comparable between diabetic and non-diabetic patients (75% vs. 74.5%, *p* = 1.00), indicating that diabetes had no measurable effect on assay performance. In contrast, ichroma IGRA-TB showed slightly higher positivity rates among non-diabetic patients (50% vs. 72.8%, *p* = 0.22), without statistical significance. Previous studies reported that diabetes increases TB susceptibility; however, evidence on its impact on IFN-γ release is inconsistent [1,2,3]. In contrast, positivity rates were significantly higher in both QFT-Plus (56% vs. 84.7%, *p* = 0.017) and ichroma IGRA-TB (48% vs. 80.4%, *p* = 0.01) among non-smokers. In this study, we found that 15 individuals tested negative in both QFT-Plus and ichroma IGRA-TB despite testing positive in culture, among whom 10 had a history of smoking and 3 were diabetic. These risk factors may impair IFN-γ-mediated immune responses and likely contributed to the reduced assay reactivity and potential decrease in sensitivity, rather than reflecting a true absence of infection, among the active TB groups (Group 3). These results are consistent with a previous study showing that tobacco smoke reduces T-cell function and IFN-γ secretion [5,6,7,8]. These results underscore the need to consider smoking status when interpreting IGRA outcomes, especially in populations with high smoking prevalence.

In this study, we observed that both QFT-Plus and ichroma IGRA-TB showed lower positivity rates among the participants with diabetes or smokers, but comparatively higher positivity rates among non-diabetic patients and non-smokers. Given the small sample size in these subgroups (smoking *n* = 25, diabetes *n* = 12), these findings are exploratory rather than conclusive. These exploratory results highlight the potential need to consider host factors, such as smoking status, when interpreting IGRA outcomes in high-risk populations.

The quantitative comparison indicated that QFT and ichroma IFN-γ assays are closely related, reflecting measurement of similar immune responses. While the assays demonstrated moderate proportional alignment, minor differences in absolute values were observed. Agreement analysis suggested that these differences are small on average, though variability exists at the individual level. Together, these findings imply that the assays capture the same underlying biological signal but are not identical in quantitative measurement. Importantly, both assays apply the same diagnostic threshold for qualitative interpretation. Despite moderate variability in absolute IFN-γ concentrations, the high qualitative agreement observed between methods indicates that clinical classification as positive or negative is largely preserved. As clinical decision-making is primarily based on threshold interpretation rather than exact quantitative values, this supports the practical applicability of the ichroma assay.

A major strength of the ichroma IGRA-TB assay is its operational simplicity. Unlike QFT-Plus, which requires trained personnel and advanced laboratory infrastructure, ichroma IGRA-TB uses a portable fluorescence reader capable of generating results within 20 min. Recent evaluations demonstrate that ichroma IGRA-TB maintains reliable IFN-γ detection with minimal technical requirements, making it suitable for decentralized TB screening [10,11]. This rapid turnaround time and ease of operation position ichroma IGRA-TB as a valuable tool for peripheral health facilities in resource-limited settings such as Bangladesh.

This study has several limitations. Firstly, we did not include any skin test as a comparator. Comparing ichroma IGRA-TB performance with available skin tests would provide additional insights into the assessment of cost-effectiveness and potential for scale-up in peripheral laboratories of Bangladesh. Secondly, we used a previous version of the analyzer, which processes one sample at a time and requires longer for a batch of 22 samples compared to QFT-Plus. The use of the next-generation AFIAS-10, which can process 10 different tests simultaneously, could resolve this issue. In addition, although comparative workflow characteristics are summarized in Table 1, we did not formally measure operator variability, failure rates, or cost per test; therefore, further studies are required to comprehensively evaluate operational performance.

## 5. Conclusions

As countries with high TB prevalence aim to reduce TB incidence, reducing the extensive LTBI reservoir is necessary to avoid recurrence; in this context, the adoption of affordable and accurate LTBI screening becomes increasingly important. This study showed a strong agreement between ichroma IGRA-TB and the more expensive QFT-Plus assay. The ichroma IGRA-TB assay could serve as a near-point-of-care test for LTBI, making it a promising option in resource-constrained settings.

## Figures and Tables

**Table 1:** Key technical and operational characteristics of QFT-Plus and ichroma IGRA-TB assay.

Features	QuantiFERON-TB Gold Plus (QFT-Plus)	ichroma^TM^ IGRA-TB
Test principle	Enzyme-linked immunosorbent assay (ELISA)	Lateral flow immunoassay (LFA)
Target analyte	Interferon-γ (IFN-γ)	Interferon-γ (IFN-γ)
TB-specific antigens	ESAT-6 and CFP-10 peptides (TB1: CD4+; TB2: CD4+/CD8+)	ESAT-6 and CFP-10 (TB Ag)
Blood collection tubes	4 tubes (Nil, TB1 Ag, TB2 Ag, mitogen)	3 tubes (Nil, TB Ag, mitogen)
Detection method	Microplate ELISA	Fluorescence-based rapid reader
Analyzer requirement	ELISA reader, washer, incubator	ichroma II portable analyzer, incubator
Turnaround time	~3 h after incubation	~20 min after incubation
Technical expertise required	Moderate to high	Low to moderate
Laboratory infrastructure	Fully equipped laboratory	Basic laboratory setting
Sample processing steps	Multiple manual steps	Fewer manual steps
Throughput	High (batch testing)	Moderate (individual testing)

**Table 2:** Demographic and clinical characteristics of enrolled participants visiting TBSTC between 15 December 2024 and 25 August 2025.

Characteristics	Category	Group 1 (*n* = 130), %	Group 2 (*n* = 70), %	Group 3 (*n* = 80), %	Group 4 (*n* = 20), %	Overall (*n* = 300), %
Sex	Female	77 (59.2)	45 (64.3)	29 (36.2)	11 (55.0)	162 (54.0)
	Male	53 (40.8)	25 (35.7)	51 (63.8)	9 (45.0)	138 (46.0)
Age group	<5	-	2 (2.9)	-	-	2 (0.7)
	6–17	5 (3.8)	12 (17.1)	4 (5.0)	1 (5.0)	22 (7.3)
	18–25	48 (36.9)	18 (25.7)	23 (28.8)	3 (15.0)	92 (30.7)
	26–35	58 (44.6)	25 (35.7)	17 (21.2)	5 (25.0)	105 (35.0)
	36–50	19 (14.6)	11 (15.7)	15 (18.8)	8 (40.0)	53 (17.7)
	>50		2 (2.9)	21 (26.2)	3 (15.0)	26 (8.7)
Diabetic	Diabetic	1 (0.8)	-	14 (17.5)	4 (20.0)	19 (6.3)
	Non-diabetic	129 (99.2)	70 (100.0)	66 (82.5)	16 (80.0)	281 (93.7)
Smoker	Non-smoker	114 (87.7)	59 (84.3)	55 (68.8)	14 (70.0)	242 (80.7)
	Smoker	16 (12.3)	11 (15.7)	25 (31.2)	6 (30.0)	58 (19.3)

**Table 3:** Distribution of QFT-Plus and ichroma IGRA-TB results among enrolled participants.

Test	Result	Group 1 (*n* = 130)	Group 2 (*n* = 70)	Group 3 (*n* = 80)	Group 4 (*n* = 20)	Overall (*n* = 300)
QFT-Plus	Positive	29 (22.3%)	22 (31.4%)	56 (70.0%)	12 (60.0%)	119 (39.7%)
	Negative	101 (77.7%)	48 (68.6%)	19 (23.8%)	8 (40.0%)	176 (58.7%)
	Indeterminate	-	-	5 (6.2%)	-	5 (1.6%)
ichroma IGRA-TB	Positive	30 (23.1%)	23 (32.9%)	52 (65.0%)	14 (70.0%)	119 (39.7%)
	Negative	100 (76.9%)	47 (67.1%)	28 (35.0%)	6 (30.0%)	181 (60.3%)
*p*-value		1.00	1.00	0.34	0.5	1.0

**Table 4:** Comparison of ichroma IGRA-TB with QFT-Plus among enrolled participants.

Group	ichroma IGRA-TB	QFT-Plus		PPA (%) (95% CI)	NPA (%) (95% CI)	Overall Agreement (%) (95% CI)	Kappa
		Positive	Negative				
Group 1 (Healthy Control)	Positive	25	5	86.2 (69.4–94.5)	95.0 (88.9–97.9)	93.1 (87.4–96.3)	0.80
	Negative	4	96				
Group 2 (TB contacts)	Positive	21	2	95.5 (78.2–99.2)	95.8 (86.0–98.8)	95.7 (88.1–98.5)	0.90
	Negative	1	46				
Group 3 (Active TB)	Positive	49	3	87.5 (76.4–93.8)	84.2 (62.4–94.5)	86.7 (77.2–92.6)	0.67
	Negative	7	16				
Group 4 (TB history)	Positive	12	2	100.0 (75.8–100.0)	75.0 (40.9–92.9)	90.0 (69.9–97.2)	0.78
	Negative	0	6				
Overall	Positive	107	12	89.9 (83.2–94.1)	93.2 (88.5–96.1)	91.9 (88.2–94.5)	0.83
	Negative	12	164				

PPA, positive percentage agreement; NPA, negative percentage agreement.

**Table 5:** Comparison of ichroma IGRA-TB and QFT-Plus with culture results.

IGRA Assay		Culture		Sensitivity (%) (95% CI)	PPV (%) (95% CI)	Accuracy (%) (95% CI)
	Result	Positive (*n* = 71)	Negative (*n* = 4)			
QFT Plus	Positive (*n* = 56)	53	3	74.6 (62.92–84.23)	94.6 (90.80–96.93)	72 (60.44–81.76)
	Negative (*n* = 19)	18	1			
ichroma IGRA-TB	Positive (*n* = 52)	49	3	69.0 (56.92–79.46)	94.2 (90.08–96.71)	66.7 (54.83–77.14)
	Negative (*n* = 23)	22	1			

PPV, positive predictive value.

**Table 6:** Association of diabetes and smoking status on assay performance of QFT-Plus and ichroma IGRA-TB among culture-positive active TB individuals.

IGRA Assay		Culture Positive (*n* = 71)			
	Result	Diabetic (*n* = 12); %	Non-Diabetic (*n* = 59); %	Smoker (*n* = 25); %	Non-Smoker (*n* = 46); %
QFT Plus	Positive (*n* = 53)	9 (75)	44 (74.5)	14 (56)	39 (84.7)
	Negative (*n* = 18)	3 (25)	15 (25.4)	11 (44)	7 (15.2)
	*p* -value	1.00		0.017	
ichroma IGRA-TB	Positive (*n* = 49)	6 (50)	43 (72.8)	12 (48)	37 (80.4)
	Negative (*n* = 22)	6 (50)	16 (27.2)	13 (52)	9 (19.5)
	*p* -value	0.22		0.01	

## Data Availability

The original contributions presented in this study are included in the article. Further inquiries can be directed to the corresponding author.

## Abbreviations

The following abbreviations are used in this manuscript:

IGRA	Interferon-gamma release assay
ELISA	Enzyme-linked immunosorbent assay
LFA	Lateral flow immunoassay
TST	Tuberculin skin test
